# 

**Published:** 1893-05-20

**Authors:** 


### CONTENTS.

**Some Neglected Medical Obligations** - - - - - 113

**A Co-operative Tour to Rome** - - - - - - - 114

**Medical History from the Earliest Times.** By E. T. WITHINGTON, M.A., M.B.Oxon. LV.—The Chemical School of Medicine - - - - - - - - - - 115

**Contemporary Theory and Research** - - - - - 116

**Diet in Disease.** By Mrs. ERNEST HART. XXI.—Under-Feeding and Over-Eating (*Continued*) - - - - - - 117

**Gleanings in Science and Medicine** - - - - - 118

**Annotations.**—Death Certification and Stillborn Babies—The London's Quinquennial Appeal—Vaccination: A Suggestion for M.P.'s - - - - - - - - - - - 119

**Notes and News** - - - - - - - - - 120

**Scraps and Gleanings** - - - - - - - 128

**The Book World of Medicine and Science**

**The Student of Medicine.**—Appointments. Vacancies

**The Hospital Clinic**—

ROYAL INFIRMARY, EDINBURGH.—Some Points in the Treatment of Scirrhus of the Breast - - - - - - 121

RHINOSCOPY. II. - - - - - - - - - 122

NEW DRUGS AND PREPARATIONS.—Alumnol - - - - - 123

ON THE ALLEGED INCREASE OF CANCER. By George King, F.I.A., F.F.A., and Arthur Newsholme, M.D., M.R.C.P. - 124

NEW APPLIANCES AND THINGS MEDICAL.—Dr. Langen's Sugar of Milk - - - - - - - - - - 124

**The Institutional Workshop**—

HOSPITAL CONSTRUCTION.—Watford Cottage Hospital - - 125

PRACTICAL DEPARTMENTS.—Bed Tables - - - - - 126

FESTIVAL DINNERS.—The London Hospital—The German Hospital, Dalston—Royal Hospital for Children and Women—Hospital for Epilepsy and Paralysis - - - - - 127

CONSTRUCTION NOTES - - - - - - - - 128

### EXTRA SUPPLEMENT.—THE NURSING MIRROR.

**News from the Nursing World.**—The Nurse Dolls—Trained Nursing in Workhouses—Pity the Children—Unwilling Departures—A Wise Appointment—A Serious Warning—"The Florence Nightingale Pledge"—Another New Nurses' Home—Short Items—"The Hospital" Endowed Bed - - - - - - - - - - - lxxi

**Sanitation for Nurses.** By A SANITARY INSPECTOR. VI.—Spain - - - - - - - - - - - lxxiii

**Conscientious Disinfection.** From a Nurse's Point of View - - - - - - - - - - - - lxxiv

**Everybody's Opinion.** Sanitary Law—Measles - - - lxxv

**The Middlesex Hospital** - - - - - - - lxxv

**Metropolitan and National Nursing Association** - lxxv

**Our Examination Questions.**—The Nursing of Bronchitis lxxvi

**Appointments—Presentation** - - - - - - lxxvii

**Notes and Queries—Wants and Workers** - - - lxxvii

**For Reading to the Sick—Nursing at Southampton** lxxvii

**The Muses' Looking Glass.** Pictures.—The Royal Academy - - - - - - - - - - - lxxviii

**The Book World for Women and Nurses** - - - lxxix

